# The Impact of Hearing Loss and Hearing Aid Usage on the Visuospatial Abilities of Older Adults in a Cohort of Combined Hearing and Cognitive Impairment

**DOI:** 10.3389/fnagi.2022.785406

**Published:** 2022-02-25

**Authors:** Nattawan Utoomprurkporn, Joshua Stott, Sergi Costafreda, Doris-Eva Bamiou

**Affiliations:** ^1^Faculty of Medicine, Chulalongkorn University, Bangkok, Thailand; ^2^UCL Ear Institute, University College London, London, United Kingdom; ^3^Chula Neuroscience Center, King Chulalongkorn Memorial Hospital, Bangkok, Thailand; ^4^Division of Psychology and Language Sciences, Faculty of Brain Sciences, University College London, London, United Kingdom; ^5^Division of Psychiatry, Faculty of Brain Sciences, University College London, London, United Kingdom; ^6^NIHR Biomedical Research Centre Hearing and Deafness, London, United Kingdom

**Keywords:** hearing, hearing impaired, visuospatial, cognitive performance, older adults (50 years and above), hearing loss

## Abstract

**Introduction:**

It has been proposed that hearing loss may result in improved visuospatial abilities. The evidence for this assertion is inconsistent, and limited to studies in congenitally deaf children, despite older adults with age-related hearing loss constituting the vast majority of the hearing impaired population. We assessed visuospatial (visuoconstruction and visuospatial memory) ability in older adult hearing aid users with and without clinically significant cognitive impairment. The primary aim of the study was to determine the effect of hearing loss on visuospatial abilities.

**Method:**

Seventy-five adult hearing aid users (HA) aged over 65 were recruited, out of whom 30 had normal cognition (NC-HA), 30 had mild cognitive impairment (MCI-HA), and 15 had dementia (D-HA). The Rey Osterrieth Complex figure test (ROCFT) copy, 3 min recall and 30 min recall tests were performed to evaluate the visuoconstructional and visuospatial memory abilities of the participants.

**Results:**

There were significant differences between the ROCFT copy, 3 min recall, and 30 min recall among the three cohorts (*p* < 0.005). Compared with previously published normative data, the NC-HA performed significantly better in the ROCFT copy (*p* < 0.001), immediate recall (*p* < 0.001), and delay recall (*p* = 0.001), while the MCI-HA performed similarly to the expected norms derived from population (*p* = 0.426, *p* = 0.611, *p* = 0.697, respectively), and the D-HA performed below this norm.

**Conclusion:**

Though visuospatial abilities tend to decline when the global cognitive functioning declines, we found suggestive evidence for positive effects of age-related hearing loss on visuospatial cognitive ability. Participants with mild cognitive impairment and hearing loss, who would have been expected to perform worse than normative data, were in fact performing as well as cognitively healthy subjects without hearing loss. Visuospatial ability could be targeted when providing rehabilitation for the older adults with hearing loss.

## Introduction

Hearing impairment especially age-related hearing impairment was found to be associated with many health conditions such as physical and cognitive frailty (Sardone et al., 2021a), mild cognitive impairment and dementia (Sardone et al., 2020a), inflammation, i.e., Inflammatory food consumption (Sardone et al., 2020b) and degeneration such as retinal vessel changes (Sardone et al., 2021b).

The most concerning health consequences of hearing impairment would be cognitive decline. It has been suggested that hearing impairment can cause cognitive deterioration through various pathways such as auditory deprivation, through depression and social isolation (Lin et al., 2011). However, not all aspects of cognitive ability are found to be worse among the hearing-impaired population. Some aspects of cognitive ability may even be better in this population. This superior ability may be useful in developing an appropriate cognitive intervention for the hearing-impaired population.

The visuospatial ability of the hearing-impaired population has been studied extensively due to a belief that this population had better visuospatial ability as a compensatory mechanism for their hearing loss. For a congenital profoundly deaf population, who may rely on sign language and lipreading, visual vigilance is needed (Rudner et al., 2016). Studies have shown a trend toward better visuospatial abilities for this population, with this enhanced ability found only in the hearing-impaired signers (Wilson et al., 1997), with individuals who were not exposed to sign language performing similarly to their peers (Parasnis et al., 1996).

The majority of hearing impairment among older adults is not congenital but results from age-related hearing loss that affects up to 1 in 3 of older adults age over 65 (WHO, 2012). There is still some controversy about the visuospatial ability of this older adult population. Some previous studies found their visuospatial memory to be worse than in their normal-hearing peers (Rönnberg et al., 2014; Rudner et al., 2016). This may result from the effect of the hearing impairment toward the global memory impairment of older adults. A hearing impairment is considered to be the highest modifiable risk factor for developing dementia (Livingston et al., 2017), as it is estimated that the risk of developing dementia increases by 1.94 times with hearing impairment (Livingston et al., 2017). Therefore, when assessing the visuospatial ability of the hearing-impaired population, overall cognitive ability should always be accounted for.

We assessed visuospatial (visuoconstruction and visuospatial memory) ability on older adult hearing aid users with and without clinically significant cognitive impairment. The primary aim of the study was to determine whether older people with hearing impairment with and without global cognitive impairment may perform differently than would be expected from normative data.

## Materials and Methods

### Participants

We recruited a convenience sample of seventy-five adult hearing aid users (HA) aged over 65, out of whom 30 had normal cognition (NC-HA), 30 had mild cognitive impairment (MCI-HA), and 15 had dementia (D-HA).

The NC-HI were recruited via recruitment flyers and posters distributed in the hearing aid center at the Royal National Throat, Nose and Ear Hospital (RNTNEH), London, United Kingdom. To ensure normal cognition, only those with a General Practitioner’s Assessment of Cognition (GPCOG; Parasnis et al., 1996) score = 9 or GPCOG score = 5–8 with informant/carer interview score = 4–6 (no memory concern for the carer) were recruited (Brodaty et al., 2004).

The MCI-HI and the D-HI were recruited via clinician referral and research registry in the memory clinics at Camden and Islington NHS Foundation Trust, London, United Kingdom. The diagnosis were made according to ICD-10 diagnostic criteria. Inclusion criteria in addition to their cognitive diagnosis were audiogram pure-tone average in the speech frequency range (500–4,000 Hz) of 30 dB HL or more in the better hearing ear.

### Materials

#### The Rey Osterrieth Complex Figure Test

Rey Osterrieth Complex figure test is a standard neuropsychological assessment commonly used to assess visuoconstructive and visual recall abilities (Shin et al., 2006). The instructions of the tests were adapted to be presented visually to the participants, which is a common practice for cognitive testing in hearing impairment participants (Pye et al., 2017).

The ROCFT can be divided into two subtasks, which are the ROCFT copy and ROCFT recall tests.

#### The Rey Osterrieth Complex Figure Test Copy

This test evaluates the visuoconstructive cognitive ability to copy an abstract figure as accurately as possible within a time limit (Shin et al., 2006). The test has also been validated among the congenitally deaf native signers (the hearing impaired population with sign as their first language) (Hauser et al., 2006).

#### The Rey Osterrieth Complex Figure Test Recall

This test evaluates episodic non-verbal visual memory by recalling the complex figure previously copied as described above (Shin et al., 2006). The participants were not made aware of the recall part when they were performing the ROCFT copy.

The recall had two stages. After 3 min from the ROCFT copy, the participant was asked to draw the figure again. The researcher took away the drawing and continued to have a conversation with the participant about their hearing problems. At 30 min, the participant was asked to draw the figure again from memory.

#### Montreal Cognitive Assessment

Montreal Cognitive Assessment is a commonly used cognitive screening test in general clinical settings. Previous research has shown that using MoCA among the hearing impaired population may result in lower score by up to −1.66 points (Utoomprurkporn et al., 2020b). This may potentially be due to the misheard of the target words of the hearing impaired population in the memory recall section of MoCA (Utoomprurkporn et al., 2020b).

Therefore, we opted for the visually based MoCA to accurately assess the cognitive abilities of the hearing impaired participants. The detailed description of the test instruction and materials can be found in our previously published paper (Utoomprurkporn et al., 2021b).

### Statistical Analysis

The Rey Osterrieth Complex figure test (ROCFT) copy, 3 min recall and 30 min recall tests were performed to evaluate the participants’ visuoconstructional and visuospatial memory abilities. The total performance scores of each test were calculated. Due to the floor and ceiling effects of the performance scores in selected cohorts, the data did not conform with normal distribution. Therefore, non-parametric statistical analysis was used. The comparison of total performance scores for each cohort was done with independent-samples Kruskal–Wallis test.

The scores were also compared to the norms for these tests, obtained from samples with normal cognition and hearing previously published for different age groups (Chiulli et al., 1995). The comparisons were done with independent-sample *t*-test.

## Results

The baseline characteristic of the three cohorts was shown in Table 1. There were significant different in the baseline characteristics of the three cohorts with the difference in the MoCA scores for each cohort were to be expected.

**TABLE 1:** Baseline characteristics of the cohorts.

Baseline characteristics	NC-HI (*n* = 30)	MCI-HI (*n* = 30)	D-HI (*n* = 15)	*P* value
Age (Mean, SD)	75.27 (SD = 5.88)	83.80 (SD = 6.42)	80.80 (SD = 8.53)	<0.0005
Education years	16.07 (SD = 3.69)	13.27 (SD = 4.17)	10.53 (SD = 3.87)	<0.0005
MoCA score	27.27 (SD = 2.16)	22.03 (SD = 3.06)	15.20 (SD = 4.21)	<0.0005

**FIGURE 1:**
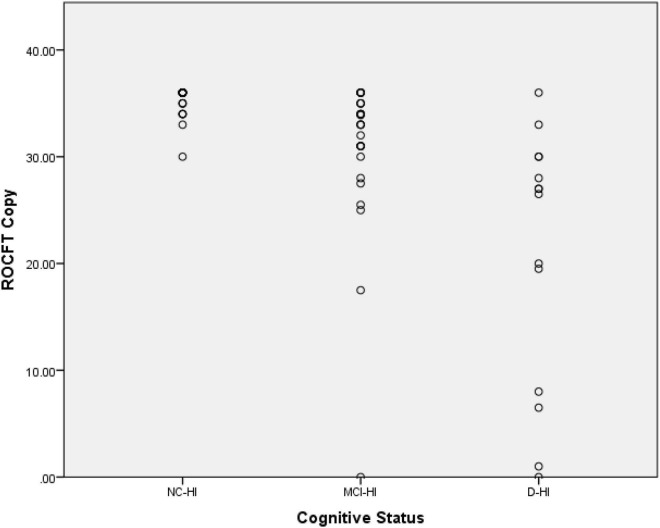
Scatter plot for the ROCFT copy scores of the three cohorts.

There were significant differences between the ROCFT copy, 3 min recall, and 30 min recall among the three cohorts (*p* < 0.005). For the ROCFT copy, the NC-HA scored 35.33 (SD = 0.24), the MCI-HA scored 31.01 (SD = 1.35), the D-HA scored 20.89 (SD = 3.24) as demonstrated in Figure 1. A slight ceiling effect was seen for the NC-HI score whereby participants reached the full score of 36.

**FIGURE 2:**
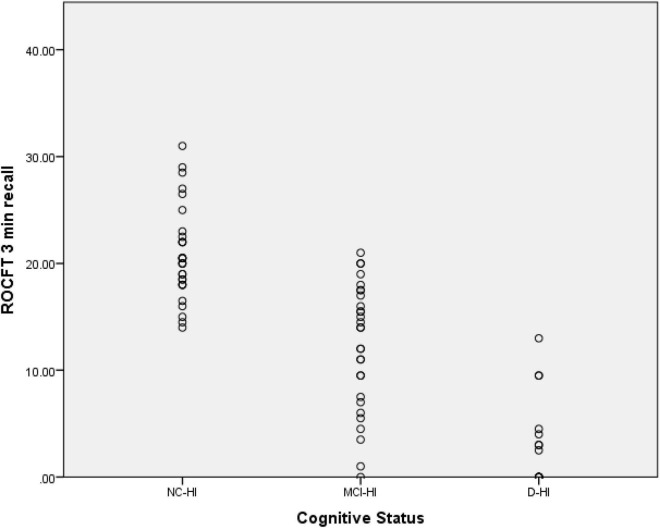
Scatter plot for the ROCFT 3 min recall scores of the three cohorts.

For the ROCFT 3 min recall; the NC-HA scored 20.85 (SD = 0.78), the MCI-HA scored 12.22 (SD = 1.09), the D-HA scored 3.50 (SD = 1.14) as demonstrated in Figure 2.

**FIGURE 3:**
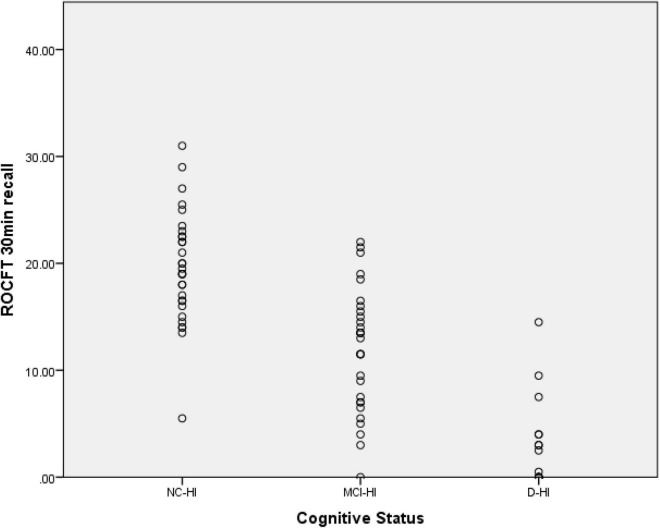
Scatter plot for the ROCFT 30 min recall scores of the three cohorts.

For the ROCFT 30 min recall; the NC-HA scored 19.63 (SD = 0.95), the MCI-HA scored 11.91 (SD = 1.07), the D-HA scored 3.46 (SD = 1.16) as demonstrated in Figure 3. A slight floor effect was seen for the D-HI score whereby some participants could not recall any part of the ROCFT figure resulting in the 0 score.

The previously published age-appropriate normative data (Chiulli et al., 1995) were also shown below in Table 2. Independent student *t*-test showed the NC-HA performed significantly better in the ROCFT copy (*p* < 0.001), immediate recall (*p* < 0.001), and delay recall (*p* = 0.001), while the MCI-HA performed similarly to the expected norms derived from the population (Chiulli et al., 1995) (*p* = 0.426, *p* = 0.611, *p* = 0.697, respectively), and the D-HA performed significantly below this norm as illustrated in Table 2.

**TABLE 2:** ROCFT scores comparison with normative data (Chiulli et al., 1995).

	Normal cognition with hearing impairment (NC-HI)	*P* value	Normative for older adults aged 70–79 years	Mild cognitive impairment with hearing impairment (MCI-HI)	*P* value	Dementia with hearing impairment (D-HI)	*P* value	Normative for older adults aged 80–91 years
ROCFT copy	35.33 (SD = 1.32)	<0.0005	31.7 (SD = 3.6)	31.01 (SD = 7.42)	0.426	20.89 (SD = 3.24)	<0.0005	29.8 (SD = 4.6)
ROCFT immediate recall	20.85 (SD = 4.29)	<0.0005	15.5 (SD = 6.6)	12.16 (SD = 5.99)	0.611	3.50 (SD = 1.14)	<0.0005	12.9 (SD = 6.4)
ROCFT 30 min recall	19.63 (SD = 5.20)	0.001	15.4 (SD = 6.4)	11.86 (SD = 5.87)	0.697	3.46 (SD = 1.16)	<0.0005	12.4 (SD = 6.0)

## Discussion

We found that cognitively healthy participants with hearing loss had significantly better visuospatial performance than expected relative to normative data from people without hearing impairment. Participants with mild cognitive impairment and hearing loss, who would have been expected to perform worse than normative data, appeared to be performing at a similar level to cognitively healthy subjects without hearing loss. Our findings suggest that age-related hearing loss/hearing aid wearers may result in improved visuospatial abilities.

However, the suggestion that visuospatial abilities outperform the global cognitive functioning is highly dependent on whether the normative data are applicable for the tested persons. Therefore, we selected the age matched published data set for this comparison. However, ideally like in many other studies on cognitive functioning, we should have built a data set of control participants within our own research and environment setting. This would be the most suitable way to clearly demonstrate the intended results.

### Rey Osterrieth Complex Figure Test Copy

The ability to copy a complex pattern such as the ROCFT reflects the participants executive functions especially planning and organizing (Shorr et al., 1992; Shin et al., 2003) and visuospatial skills. For the NC-HI, their visuospatial performances were significantly better than the norms even though research showed a decrease overall cognitive ability in this population (Utoomprurkporn et al., 2020a). This may be because the assessment mode of most commonly used cognitive tools is auditory based, which may hinder the hearing-impaired population responses. Therefore, our result suggested that visually based cognitive screening tools may also be considered when assessing the hearing-impaired population ability (Utoomprurkporn et al., 2021b). It may unveil their compensated cognitive ability via other means such as visuospatial found in our cohort.

Since we targeted hearing aid wearers, this improved visuospatial may also be a result of hearing aid use. Previous UK Biobank research showed that hearing aid acted as a protective factor against visuospatial working memory error despite decreasing overall working memory of the hearing-impaired population (Rönnberg et al., 2014).

For the MCI-HI, the ROCFT copy ability, though decreased from the N-HI, still performed comparably to the norms. Although memory is the first function affected in most individuals with a MCI diagnosis, their executive functions are also commonly impaired (Traykov et al., 2007). Still, the impairment in executive function is usually mild since, by definition of MCI, activities of daily life should not be affected by the impairment (Petersen, 2004). When the activity of daily life is affected, by definition, these individuals would be in early stages of dementia. Therefore, as expected, we found a relatively smaller mean difference score in the ROCFT copy task between the NC-HI and the MCI-HI versus between the MCI-HI and the D-HI.

### Rey Osterrieth Complex Figure Test Recall

Several studies have shown that older adults with hearing loss may have worse episodic memory of target words than their normal-hearing peers even after accounting for cognitive status (Lim and Loo, 2018). However, most studies were done with verbally administered target words. Therefore, the participants may not recall the word due to encoding problems (i.e., because they misheard the target words or due to fatigability from listening to the unclear target words). It is crucial to understand what causes the observed impairment in episodic memory among the hearing-impaired population since this is a hallmark for Alzheimer’s type dementia (Thompson et al., 2005).

Visually based assessments were previously found to measure the hearing impairment population’s cognitive abilities more accurately than verbal assessment (Wong et al., 2019). The ROCFT recall does not tap into the language/auditory memory of the participant, which may be affected by hearing impairment.

For the MCI population, memory ability, as assessed in ROCFT recall is usually impaired early in the disease progression (Jessen et al., 2014). However, for the MCI-HI, the visuospatial memory ability, though decreased from the N-HI, still performed comparably to the norms. This finding should be considered when evaluating the hearing-impaired population for early signs of MCI via visual assessment. They may have a comparable result with the norms despite fulling the diagnostic criteria of MCI.

We found a relatively larger mean difference score in the ROCFT recall task, which reflects the memory domain, than in the mean difference in the ROCFT copy tasks among the three cohorts. This may be because the memory impairment starts earlier with a larger effect in the course of MCI and dementia disease progression. The ROCFT recall tasks were significantly worse among D-HI and MCI-HI compared with the NC-HI group in both in the 3 min recall and the 30 min recall tasks.

### Possible Explanations for Improved Visuospatial Ability

Previous research showed similar trends that individuals who needed to utilize lipreading and sign language, i.e., the early onset or the profound hearing impairment population, may have better visuospatial abilities than their normal-hearing peers (Bavelier et al., 2006; Rudner et al., 2016). Neuroimaging studies showed evidence of cross-modal plasticity among the hearing-impaired population, and early adoption of sign language could enhance this process (Simon et al., 2020). Even though we specifically excluded the populations with childhood early-onset hearing impairment and only targeted hearing-impaired older adults hearing aid users who are not as severe to be on the cochlear implant waiting lists to avoid this potential confounder, we still found better visuospatial ability among our NC-HI cohort.

Currently, several United Kingdom local support groups for hearing aid users provide various kinds of support, including manipulating the hearing devices, psychological support, and lipreading and facial cue interpretation lessons. Several participants in our cohorts were also active participants in these other supporting activities. Therefore, this may be another potential explanation for their superior visuospatial abilities.

Lipreading is generally used for communication purposes by hearing-impaired individuals. Even without hearing aids, the speech recognition scores of hearing-impaired individuals improve with lipreading (Dell’Aringa et al., 2007). It is estimated that lipreading alone can help listeners catch about 50% of the conversation. In combination with hearing aids, the older adults’ ability to understand speech may increase by up to 93.5%. These findings may indicate that the older hearing-impaired population also benefit from superior visuospatial ability through lipreading in everyday situations, despite not using sign language.

Our research also indicates that the cross-modal plasticity may not only present in the congenital deaf population. It may also occur later in life in older adults with age-related hearing loss who usually start developing hearing impairment in middle age (World Health Organization, 2021). With cross-modal plasticity, previous research suggested an inverse relationship between improved visual abilities and speech recognition (Glick and Sharma, 2017; Gilissen and Arckens, 2021). We also found that our dementia cohort tend to report lower speech recognition and understanding (Utoomprurkporn et al., 2021a). However, further research is needed to explore this inverse effect in more details. Interestingly, previous research also suggested that hearing intervention can create positive changes in the cortical plasticity (Glick and Sharma, 2017). This effect should be further studied to improve the quality of life for these older adults with hearing impairments.

Many studies among older adults who underwent auditory interventions with cochlear implantations also showed improvement in overall global cognition indicating positive cortical plasticity despite their older ages (Mosnier et al., 2015; Cosetti et al., 2016). However, these individuals may not be able perform on par with their peers therefore additional cognitive training may be needed to enhance their rehabilitation program (Claes et al., 2018; Mertens et al., 2020). It is noted that when the subject’s visuospatial abilities were evaluated as part of a subdomain of a cognitive assessment composite test, their superior visuospatial ability may not always be apparent.

This superior visuospatial ability could be of benefit even in the presence of global deterioration of overall cognitive function in hearing impaired individuals. Further rehabilitation program may utilize lipreading techniques and use of other visuospatial clues such as facial expression, etc. By emphasizing the communication techniques that are most accessible to them, these individuals would have better communication and would potentially preserve longer their cognition. A previous systematic review also showed the importance of communication strategies and techniques in addition to hearing amplification for this group of older adults with hearing impairments (Hawkins, 2005).

Another potential explanation is the sampling issue. We did not have a control group where the participants did not have hearing impairment. We had to compare the data to the previously published norm. The general health and education levels of our participants may differ from those in the norm samples. We did not have the information about the norms overall cognitive functioning data such as IQ, etc. Therefore, there is a possibility that our NC-HI cohorts may had higher overall functioning than the normative data set, also reflected in the superior ROCFT scores. Further study to compare these cohorts with normal hearing controls, matched for IQ may be needed to investigate this aspect in more detail.

## Limitation

The main limitation of our study is that we had to compare the data to the normative data study that was conducted in 1995 as we did not have a control group with normal hearing. Moreover, the age and education years were slightly different among the cognitively normal and cognitively impaired cohorts. This discrepancy and its implications of it were discussed and explored more in our previous published work (Utoomprurkporn et al., 2021b).

With a relatively small sample size, the lack of difference of ROCFT scores for the MCI-HI and the D-HI compared with norms could also be due to inadequate statistical power. Further studies with larger cohorts of cognitively impaired older adults hearing aid users may be useful to explore their ability in more detail.

## Implication/Conclusion

We found suggestive evidence for the positive effects of age-related hearing loss on visuospatial cognitive ability. The visuospatial ability could be targeted when providing rehabilitation for the older adult with hearing loss, for example, with lipreading training, to support their communication.

## Data Availability Statement

The raw data supporting the conclusions of this article will be made available by the authors, without undue reservation.

## Author Contributions

NU, D-EB, JS, and SC contributed to the conception and design of the study. NU organized the data collection and database, performed the statistical analysis, and wrote the first draft of the manuscript. All authors contributed to manuscript revision, read, and approved the submitted version.

## Conflict of Interest

The authors declare that the research was conducted in the absence of any commercial or financial relationships that could be construed as a potential conflict of interest.

## Publisher’s Note

All claims expressed in this article are solely those of the authors and do not necessarily represent those of their affiliated organizations, or those of the publisher, the editors and the reviewers. Any product that may be evaluated in this article, or claim that may be made by its manufacturer, is not guaranteed or endorsed by the publisher.
